# Correlations between specific patterns of spontaneous activity and stimulation efficiency in degenerated retina

**DOI:** 10.1371/journal.pone.0190048

**Published:** 2017-12-27

**Authors:** Christine Haselier, Sonia Biswas, Sarah Rösch, Gabriele Thumann, Frank Müller, Peter Walter

**Affiliations:** 1 Department of Ophthalmology, RWTH Aachen University, Aachen, Germany; 2 Institute of Complex Systems, Cellular Biophysics, ICS-4, Forschungszentrum Jülich GmbH, Jülich, Germany; University of California Berkeley, UNITED STATES

## Abstract

Retinal prostheses that are currently used to restore vision in patients suffering from retinal degeneration are not adjusted to the changes occurring during the remodeling process of the retina. Recent studies revealed abnormal rhythmic activity in the retina of genetic mouse models of retinitis pigmentosa. Here we describe this abnormal activity also in a pharmacologically-induced (MNU) mouse model of retinal degeneration. To investigate how this abnormal activity affects the excitability of retinal ganglion cells, we recorded the electrical activity from whole mounted retinas of *rd10* mice and MNU-treated mice using a microelectrode array system and applied biphasic current pulses of different amplitude and duration to stimulate ganglion cells electrically. We show that the electrical stimulation efficiency is strongly reduced in degenerated retinas, in particular when abnormal activity such as oscillations and rhythmic firing of bursts of action potentials can be observed. Using a prestimulus pulse sequence, we could abolish rhythmic retinal activity. Under these conditions, the stimulation efficiency was enhanced in a few cases but not in the majority of tested cells. Nevertheless, this approach supports the idea that modified stimulation protocols could help to improve the efficiency of retinal prostheses in the future.

## Introduction

Photoreceptor degenerations such as retinitis pigmentosa (RP) or others are often inherited diseases and the progressive loss of rods and cones in the outer retina lead to untreatable blindness. The inner retina undergoes a remodeling process but most retinal neurons, in particular the retinal ganglion cells (RGCs), are preserved [1–3]. Thus, retinal prostheses represent one approach to restore vision in blind patients. The currently applied concepts of electrical retinal stimulation using implantable electrode arrays do not take into account that remodeling processes are occurring during the degeneration process in the inner retina and that they may cause changes in electrophysiological properties of the retina. [4–6]

The *rd10* mouse is an appropriate animal model for RP. A missense mutation in the gene for the rod phosphodiesterase β subunit results in rod degeneration [7, 8]. Rod degeneration starts around postnatal day 18 (P18) when all retinal connections have been established and is followed by cone degeneration [9, 10]. Retinal remodeling encompasses retraction of rod bipolar cell and horizontal cell dendrites as well as extension of Müller cells leading to gliosis [11]. The persisting RGCs represent a target for electrical stimulation by retinal prostheses to elicit visual percepts [6]. However, the RGC activity differs between *rd10* retina and wild type retina. The spontaneous firing rate of *rd10* RGCs is increased and sometimes action potentials appear as bursts phase locked with slow oscillatory potentials. Although the origin of these oscillations has not been fully elucidated, it has been suggested that in both *rd1* and *rd10*, oscillations are generated by electrically coupled cone ON bipolar cells and AII amacrine cells. [12–16]. However, not all of these changes seem to directly depend on photoreceptor death because hyperactivity in *rd10* emerges before rod degeneration starts [13, 15].

Photoreceptor degeneration can also be induced pharmacologically. In previous studies it was shown that intraperitoneally injected N-Methyl-N-nitrosourea (MNU) leads to specific photoreceptor death by apoptosis [17–20]. Similar to the genetic *rd10* model, in MNU-treated retinas remodeling of the inner retina is observed while the RGCs remain unaffected up to three months after injection [18, 19].

In this study, we compare the spontaneous activity of RGCs between wild type mouse retina, *rd10* mouse retina, and MNU-treated retina. We show spontaneous rhythmic activity in MNU-treated retina similar to that found in *rd10* retina. Furthermore, we show that irrespective of the type of degeneration the efficiency to drive RGC activity by electrical stimulation is much lower in the degenerated retina compared to wild type retina. These effects must be considered when algorithms for retinal stimulation in clinical situations are discussed and defined.

## Materials and methods

All experiments were carried out in accordance with the ARVO declaration for the use of animals in ophthalmic and vision research, in accordance with the German Law for the Protection of Animals and after permission by the regulatory authorities, the Institute for Laboratory Animal Science of the University Hospital RWTH Aachen and the Landesamt für Natur, Umwelt und Verbraucherschutz of the land North Rhine-Westphalia (84–02.04.2011.A386) specifically approving this study.

### MNU treatment

A detailed description of MNU injections and its effects on the morphology of the retina was given by Rösch et al. [20]. In brief, adult (8–16 weeks) C57BL/6J mice received intraperitoneal injections of 60 mg/kg BW MNU (Sigma Aldrich, Germany). The studies of Nambu et al. and Rösch et al. showed that 7 days after MNU treatment the outer nuclear layer was lost or massively reduced [17, 20]. Additionally, we performed spectral domain optical coherence tomography (OCT) (Heidelberg Engineering, Heidelberg, Germany) scans 9 days after injection to ensure the absence of photoreceptors (data not shown). Animals were euthanized and the retinas were isolated as described below 9, 10, and 11 days after injection.

### Tissue preparation

After Euthanasia by an overdose of isoflurane (Forene 100% [vol/vol]; Abbott GmbH, Wiesbaden, Germany) and decapitation, eyes of *rd10* and C57BL/6J (wild type) mice of post natal week (PNW) 2, 4, 12, 20 as well as eyes of MNU-treated wild type mice were enucleated and transferred into oxygenated AMES solution (Sigma Aldrich, Germany). The eye was opened along the ora serrata, the lens and vitreous were removed. The retina was separated from the eyecup and was flattened with the photoreceptor side on a piece of nitrocellulose with a precut window. After removing of remaining fluid the retina was placed with the ganglion cell side down onto the 60 TiN electrodes of a planar 8x8 multi electrode array (MEA) with an electrode spacing of 200 μm and an electrode diameter of 30 μm (Multichannel Systems, Reutlingen, Germany). MEAs were pretreated with a plasma cleaner (Diener Electronic, Ebhausen, Germany) and coated with 0.25 mg/ml poly-D-lysine hydrobromide for about 2 h (Sigma Aldrich, Germany).

### MEA recordings and electrical stimulation

The MEA2100-system (Multichannel Systems, Reutlingen, Germany) was used for recording and electrical stimulation. During all measurements the retina was perfused with oxygenated AMES solution (carbonate buffered to pH 7.4) with a rate of 3 ml/min at room temperature. For electrical stimulation one of the electrodes was defined as stimulation electrode and the others were used for recording. To determine the stimulation efficiency in retinas of wild type, *rd10* and MNU-treated mice, single biphasic current pulses of different amplitudes (30, 60, 80, 90 μA) and durations (500, 1000 μs) were applied with the cathodic phase first. Results were obtained from 80–120 cells, recorded from five retinas of at least three animals for each stimulus setting. For the prestimulus pulse sequence experiments, retinas of *rd10* and MNU-treated mice were stimulated with a multi (10x) biphasic current pulse of 80 μA and 1000 μs with a frequency of 1 Hz first. When oscillations and bursting disappeared, a single biphasic pulse with the same amplitude and duration was applied. With MC-Rack Software (Multichannel Systems, Reutlingen, Germany) data were recorded with a sampling rate of 25 kHz and stimulation parameters were programmed.

### Analysis

Data were filtered either with a band pass filter of 100–3000 Hz (to isolate spikes) or with a low pass filter of 50 Hz (to isolate oscillations). Using the MC-Data Tool Software (Multichannel Systems, Reutlingen, Germany) data obtained with MC-Rack were transformed to ASCII files for further analysis with Origin (Microcal Software, Northampton, USA) or MATLAB (MathWorks, Natick, USA). To compare the stimulation efficiency the spike rate ratio was calculated. To obtain the spike rate ratio, the spike rate recorded in a time window of 400 ms after stimulation was divided by the spike rate measured in an 8 sec long time window before the stimulation. Spike rates were determined by Neuroexplorer (Nex Technologies, Madison, USA). Spike sorting was carried out with Offline Sorter (Plexon Inc, Dallas, USA). Sorting criteria for the categories oscillation vs. no oscillation, rhythmically occurring bursts vs. no bursts: we analyzed a period of at least 8 seconds before the stimulus pulse. In most cases, oscillations were prominent enough to be identified by eye. If not, sorting was supported by Fast Fourier analysis. We defined cells as rhythmically bursting units, when we observed groups of at least two action potentials that appeared in a rhythmic manner. In those cases, in which bursts were observed in the presence of oscillations, bursts were typically phase-locked to the oscillations. In inter spike interval analysis of rhythmically bursting units, two clear peaks corresponding to the intervals within the burst and between the bursts, respectively, could be distinguished.

## Results

### Spontaneous activity is similar in MNU-treated retinas and *rd10* retinas

**Fig 1:**
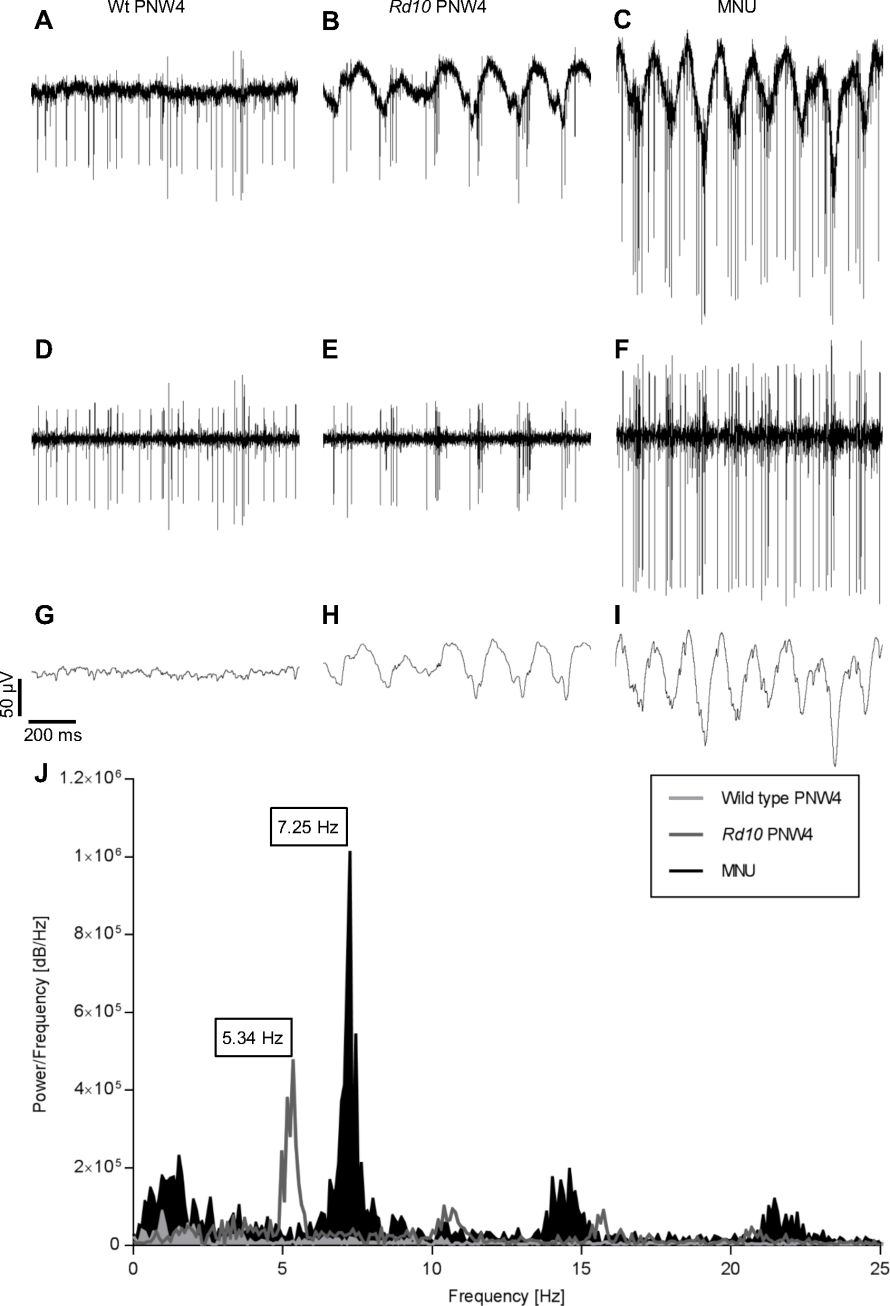
Spontaneous activity in wild type and degenerated retina. Raw data of a PNW4 wild type retina (A), a PNW4 *rd10* retina (B), and a MNU-treated retina 11 days after injection (C) and after applying a 100–3000 Hz band pass filter (D-F) or a 50 Hz low pass (G-I) filter. J: Raw data were analyzed by a fast Fourier transformation (FFT). Both types of degenerated retina showed burst activity and oscillations in contrast to the wild type retina. The FFT revealed a main peak at 5.34 Hz for the recording from *rd10* retina, at 7.25 Hz for the recording from MNU-treated retina and no peak for the recording from wild type retina. Peaks with lower amplitude at the second and third harmonic frequencies were also observed.

Previous studies have shown that spontaneous activity differs between the retinas of wild type and the retinas of *rd10* and *rd1* mice. In Fig 1 we compare the spontaneous activity of retinas from a wild type mouse (A, D, G), *rd10* mouse (B, E, H), and from a mouse that had received an intraperitoneal injection of MNU 11 days before the recording (C, F, I). Raw data (Fig 1A–1C) were filtered with a band pass of 100–3000 Hz (Fig 1D–1F) or with a low pass of 50 Hz (Fig 1G–1I). In wild type, continuous spiking activity (Fig 1D) and a stable baseline (Fig 1G) were observed. In contrast, in *rd10* spikes were grouped in bursts (Fig 1E) and slow oscillatory waves could be observed (Fig 1H). Interestingly, a very similar pattern of spontaneous activity was observed in recordings of MNU-treated retina (Fig 1F and 1I). With a frequency of approximately 7 Hz the oscillations in MNU treated retinas were faster than in *rd10* (approximately 5 Hz, Fig 1J, [12, 16]) but slower than in *rd1* (around 10 Hz) [12, 14, 21]. The presence of oscillatory activity seems to be a common feature in degenerated retinas, irrespective of the cause of degeneration.

**Fig 2:**
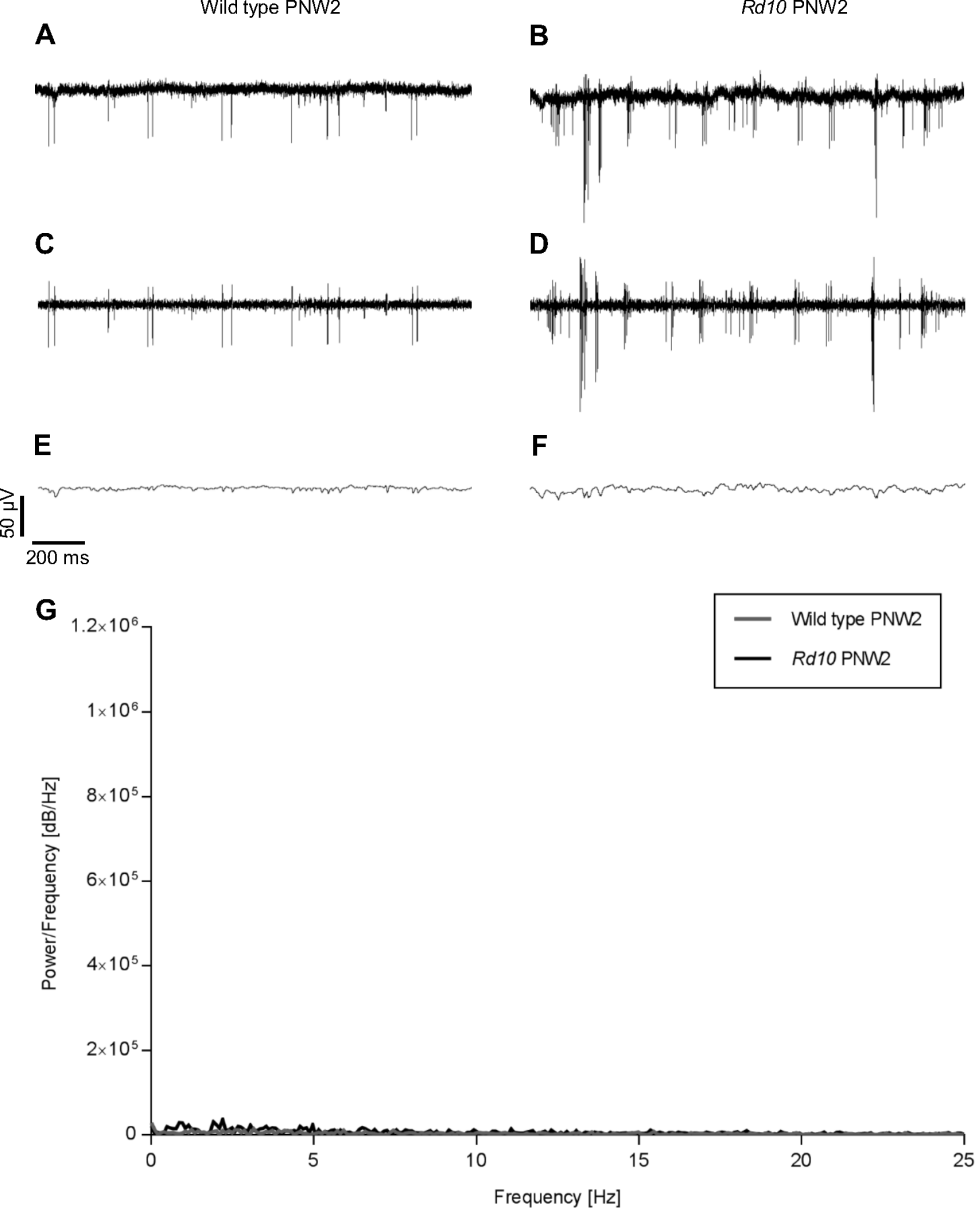
Spontaneous activity of PNW2 wild type and *rd10* retina. The raw data in A (wild type) and B (*rd10*) were band pass filtered (C, D) and low pass filtered (E, F). Spiking occurred in a stochastic manner and the baseline was stable in both wild type and *rd10*. G: No peaks were detectable in the FFT of the raw data.

In *rd10* retina, the firing pattern depended on the age of the animal. Whereas oscillations and bursts were observed from PNW4 on, at PNW2 spikes were fired in a more scattered and less rhythmic manner (Fig 2B and 2D) and no oscillations appeared (Fig 2B, 2F and 2G). The spontaneous activity of a PNW2 *rd10* retina was similar to that of the age-matched wild type retina (Fig 2A–2E and 2G).

### Electrical stimulation efficiency is lower in degenerated retina

**Fig 3:**
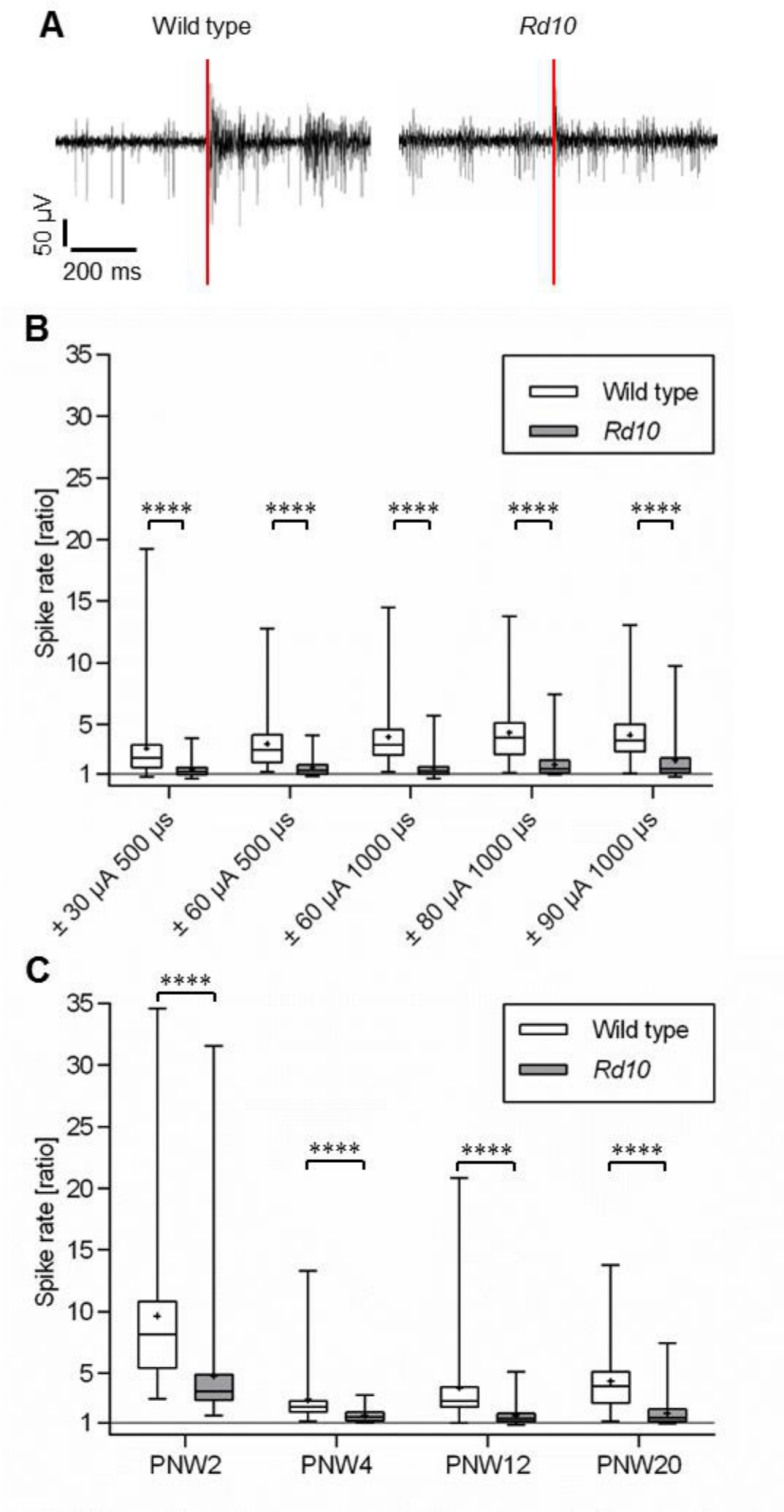
Spike rate ratio was lower in *rd10* than in wild type retina. A: Representative recordings from wild type and from *rd10* retina. B, C: The ratio of the spike rate after stimulation divided by the spike rate before stimulation (“spike rate ratio”) was determined for different stimulation parameters and in different phases of degeneration. Each column represents the median, maximum, minimum, and the 50th percentile of the data for 5 different wild type and *rd10* retinas, respectively. Each mean is marked by a cross. B: Data from PNW20 wild type and *rd10* mice are shown. The spike rate ratio was significantly higher in wild type than in *rd10* retina for all stimulation parameters. C: Data of stimulation with a single pulse of -/+ 80 μA and 1000 μs are shown. Data for PNW20 are reprinted from Fig 3B. Electrical stimulation was successful in all investigated age groups. For all age groups spike rate ratio was higher in wild type than in *rd10*. In *rd10* at PNW2, spike rate ratio was significantly higher than at PNW4, PNW12 and PNW20. In both wild type and *rd10*, the spike rate ratio did not significantly decrease further between PNW4 and PNW20. (****p ≤ 0.0001; Mann-Whitney-U-test).

We applied biphasic current pulses of different amplitude and duration to one electrode and determined the stimulation efficiency in nearby electrodes in both PNW20 wild type and *rd10* retina (Fig 3A). The cell recorded from wild type retina responded to the stimulus with long bursts of action potentials (red line indicates stimulus pulse). The unit recorded from *rd10* retina displayed typical bursts of action potentials that were independent of the stimulation. Only few additional action potentials could be observed after the stimulation pulse. In Fig 3B the spike rate ratio (spike rate after stimulation divided by spike rate before stimulation) determined at the electrodes surrounding the stimulation electrode was plotted versus the stimulation parameters (values are averaged from 5 retinas). In wild type retina the average spike rate ratio increased with stimulus amplitude and duration for the first three stimulus settings in a statistically significant way from 3.1 to 4.4. At higher stimulus settings the stimulation efficiency saturated and spike rate ratios did not differ significantly. In *rd10* retina the spike rate ratio increased only slightly from 1.3 to 2.1. The spike rate ratios were only weakly or not at all statistically different between the different stimulus settings. The average spike rate ratio was significantly lower in *rd10* than in wild type retinas for all stimulus parameters tested. Median spike rate ratios were slightly larger than 1, indicating that RGCs could be stimulated in *rd10* retina, but less efficiently than in wild type retina.

We also applied the same current pulses to retinas of different age groups. Again, the spike rate ratio was higher in wild type than in *rd10* for all age groups tested. In PNW2 *rd10* retinas, i.e. when photoreceptor degeneration had not yet started, the spike rate ratio was much higher compared to the spike rate ratio of the older *rd10* retinas. Since the spike rate ratio of PNW2 wild type retina was also higher than in retinas of older wild type mice the difference between *rd10* and wild type at PNW2 is still significant.

**Fig 4:**
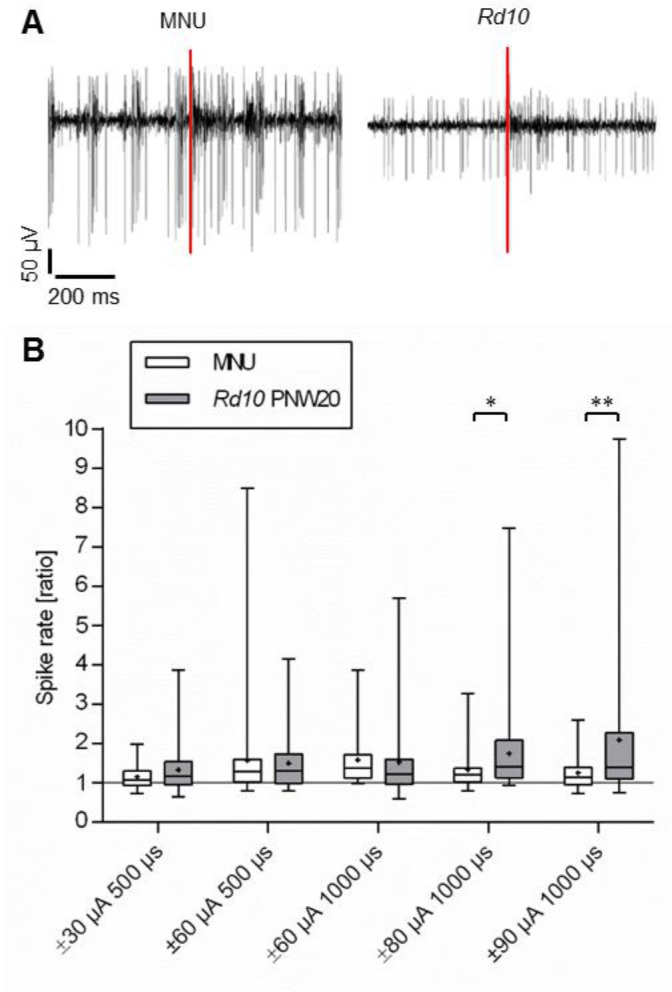
Stimulation efficiency of MNU-treated retinas. A: Representative recordings from MNU-treated retina and from *rd10* retina. B: The spike rate ratio was analyzed for different stimulation parameters. Each column represents the median, maximum, minimum, and the 50th percentile of the data for 5 different MNU-treated retinas and *rd10* retinas at PNW20, respectively. Each mean is marked by a cross. The spike rate ratio of MNU-treated retinas was comparable to that of *rd10* retinas or lower, but larger than 1, indicating that electrical stimulation was possible in MNU-treated retinas. (*p ≤ 0.05; **p ≤ 0.01; Mann-Whitney-U-test).

The stimulation efficiency in MNU-treated retinas was also reduced compared to wild type. The median spike rate ratio of 5 MNU-treated retinas was as low as the median spike rate ratio of PNW20 *rd10* retinas or even slightly lower (Fig 4).

### Stimulation efficiency depends on the pattern of spontaneous activity

**Fig 5:**
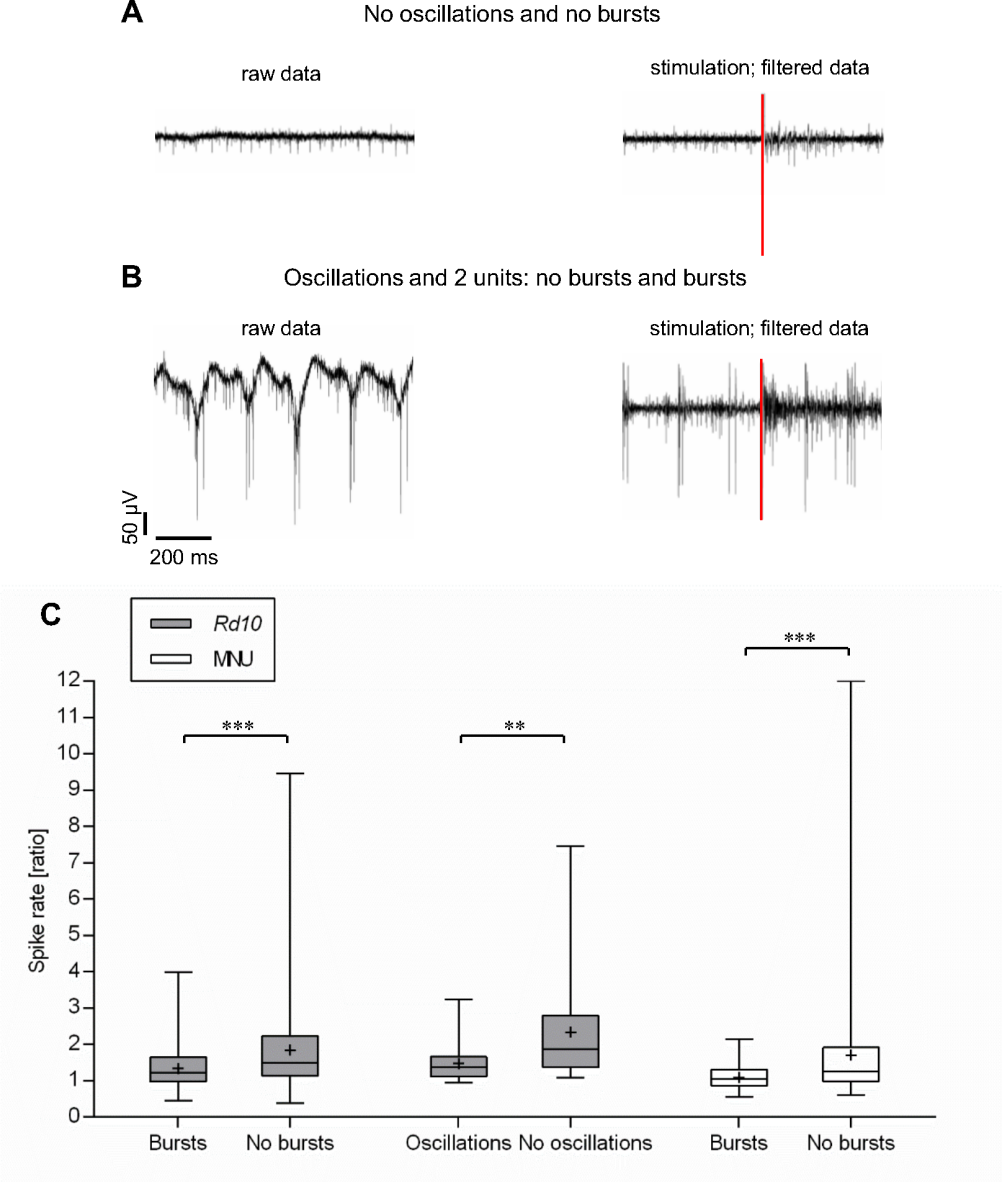
Stimulation efficiency depends on the pattern of spontaneous activity. Spontaneous activity was divided into four categories: oscillations, no oscillations, bursts and no bursts. A: representative recording for the category no oscillation–no bursts. B: representative recording for the categories oscillation and no bursts (small amplitude spikes), and oscillation and bursts (large amplitude spikes). C: The spike rate ratio of recordings from 13 *rd10* and 6 MNU retinas was analyzed (stimulation: single pulse of -/+ 80 μA and 1000 μs) and classified according to the four categories. Each column represents the median, maximum, minimum, and the 50th percentile. Each mean is marked by a cross. The spike rate ratio was significantly lower when spikes were fired in bursts (*rd10* and MNU) and in presence of oscillations (*rd10*). (**p ≤ 0.01; ***p ≤ 0.001; Mann-Whitney-U-test).

We assumed that the low stimulation efficiency observed in *rd10* retina and MNU-treated retinas could be the consequence of the spontaneous rhythmic activity observed in those retinas. To examine that, we defined four different patterns of spontaneous activity: baseline showing oscillations or no oscillations, and spikes coming in rhythmically occurring bursts (for brevity: bursts) or not in rhythmically occurring bursts (for brevity: no bursts). We categorized the recordings from each electrode according to these criteria and calculated the spike rate ratio after applying a biphasic pulse of ± 80 μA and 1000 μs. For the patterns “oscillations” and “no oscillations” we took all spikes measured by one electrode into account. For the patterns “bursts” and “no bursts” we performed a spike sorting to distinguish units firing in bursts from units not firing in bursts and calculated the spike rate ratio for each defined unit separately. Fig 5A shows recordings from a piece of retina without oscillations. The spikes of this unit are not fired in bursts (left). After the simulation, spike rate increases (right). In Fig 5B, a recording from a retina with oscillations is displayed. The spikes of two cells can be distinguished: bursts of 2–3 large amplitude spikes are phase-locked to the oscillations, while the small amplitude spikes are not fired in bursts. While the stimulus pulse triggers a burst of small amplitude spikes, the firing pattern of the large amplitude spikes remains mostly unchanged. Fig 5C shows the results obtained in 13 *rd10* retinas and in 6 MNU-treated retinas. In *rd10*, the average spike rate ratio was approximately 1.34 when spikes appeared in rhythmically occurring bursts whereas an average spike rate ratio of approximately 1.84 could be reached when spikes were fired continuously. When no oscillations were detectable the average spike rate ratio was approximately 2.34 and thus obviously higher than the obtained average spike rate ratio of approximately 1.47 when simultaneously oscillations were recorded. Similarly, the average spike rate ratio in the MNU model was significantly higher in units which showed no rhythmic burst activity (≈ 1.70) compared to units which showed rhythmic burst activity (≈ 1.09). The possible effect of oscillations on the stimulation efficiency could not be further investigated in the MNU model because the oscillations were very persistent in all MNU-treated retinas and, therefore, it was not possible to stimulate in natural absence of oscillations. Nevertheless, the stimulation efficiency depended on the pattern of spontaneous activity in both retinal degeneration models.

### Modulation of oscillations and bursts using electrical stimulation

**Fig 6:**
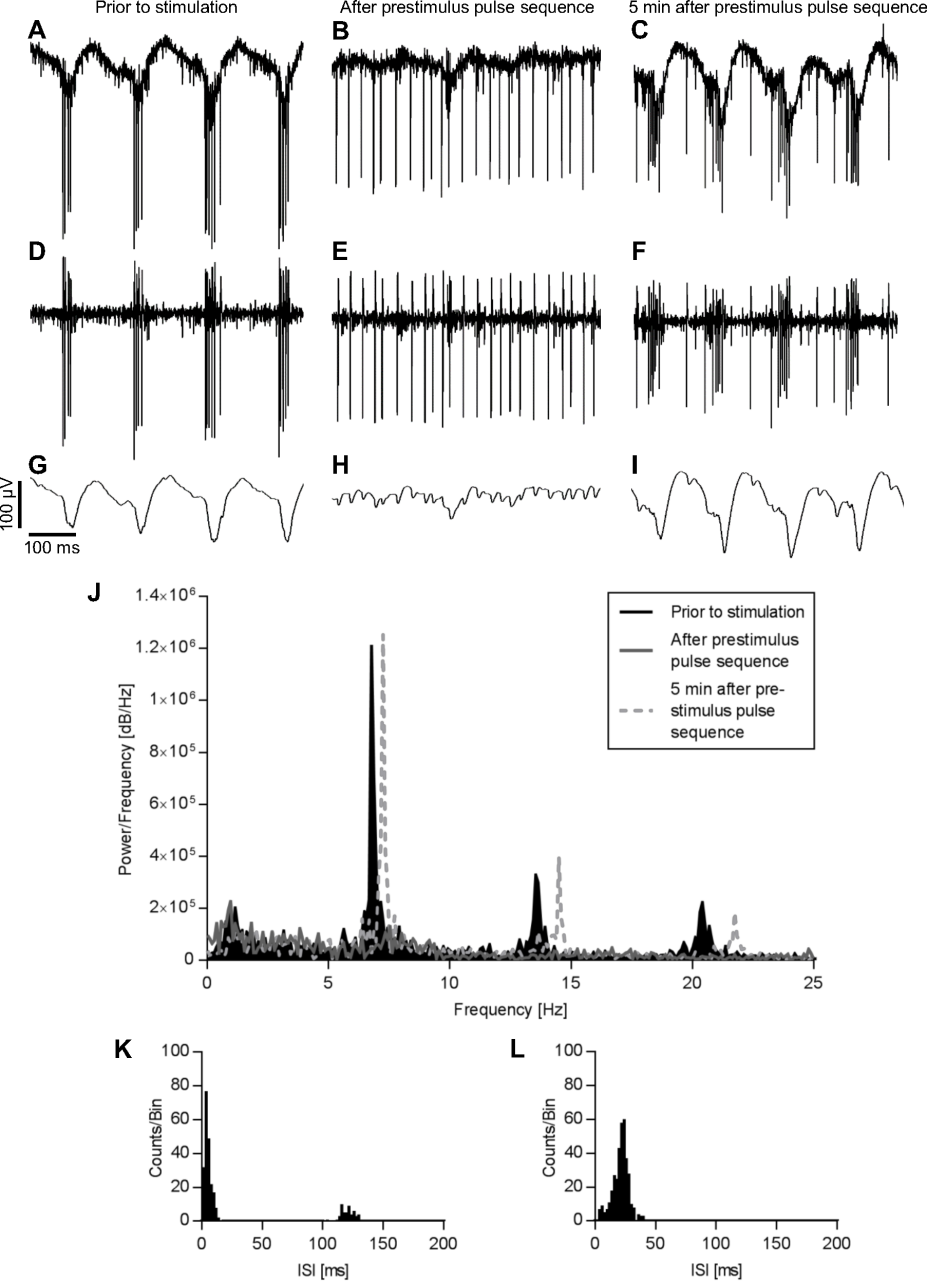
A prestimulus pulse sequence abolished oscillations and burst activity. Raw data (A-C), 100–3000 Hz band pass filtered data (D-F) and 50 Hz low pass filtered data (G-I) of an MNU-treated retina prior to (A, D, G), immediately after (B, E, H) and 5 minutes after (C, F, I) a prestimulus pulse sequence (-/+ 80 μA, 1000 μs, 10 pulses, 1 Hz). Oscillations and burst activity were abolished by the prestimulus pulse sequence but returned 5 minutes later. J: The fast Fourier analysis showed that the main peak at 7 Hz and the peaks at harmonic frequencies disappeared after the prestimulus pulse sequence and came back with a slightly increased frequency after 5 minutes. K: The interspike interval (ISI) prior to the prestimulus pulse sequence exhibited the firing of bursts with an interval of approximately 8 Hz (120 ms). After the prestimulus pulse sequence (L), the ISI revealed no bursts anymore. Bin size = 2 ms.

We recently showed that in the degenerated retina phases with pronounced oscillations can alternate with phases in which no oscillations can be observed [16]. Here, we show that a set of electrical pulses termed “prestimulus pulse sequence” can induce the switch from the oscillatory to the non-oscillatory mode. Fig 6A, 6D and 6G show a typical recording from a MNU-treated retina, displaying pronounced oscillations in conjunction with action potentials fired in rhythmically occurring bursts. After a prestimulus pulse sequence of -/+ 80 μA and 1000 μs (10 pulses, 1 Hz) applied by one electrode (distance to recording electrodes: 400 μm) the oscillations disappeared and firing became continuous (Fig 6B, 6E and 6H). In different experiments, the switch from bursting to continuous spiking occurred at slightly different timepoints in the prestimulus pulse sequence but usually happened during the second half. This effect persisted up to a few minutes after which the oscillations came back and spiking appeared in bursts again (Fig 6C, 6F and 6I). The Fourier analysis in Fig 6J confirms these observations. The peaks at approximately 7 Hz and at the harmonic frequencies vanished after application of the prestimulus pulse sequence and came back with a slightly increased frequency after 5 minutes. In the analysis of interspike intervals (ISI) two peaks corresponding to ISI within the bursts and between the bursts, respectively, were observed before the stimulus (Fig 6K). After the prestimulus pulse sequence, only one peak could be observed (Fig 6L). Similar results could be observed in 12 of 14 MNU-treated retinas. We could not observe a clear correlation between the magnitude of the response to the prestimulus pulse sequence and the efficiency at reducing oscillations.

**Fig 7:**
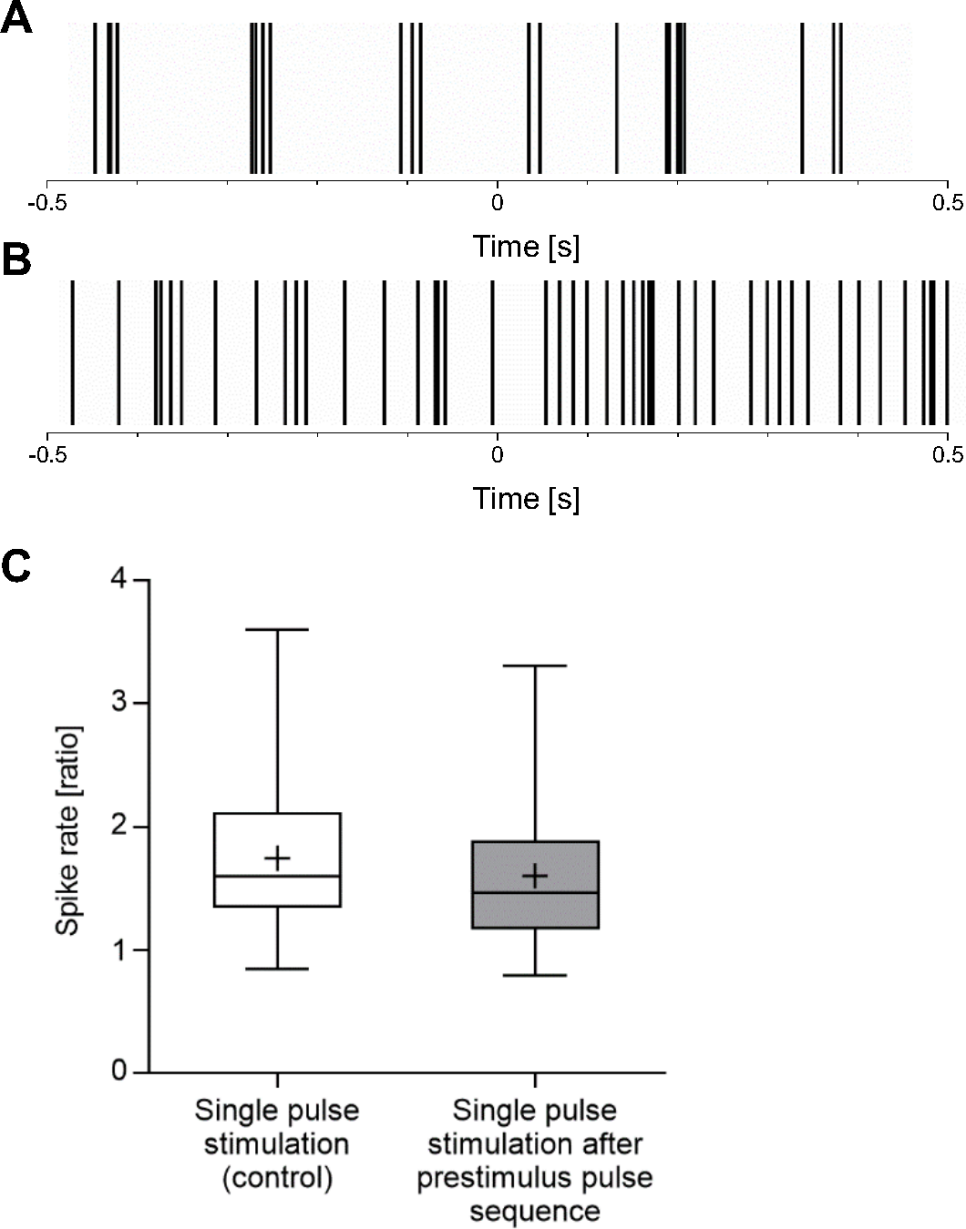
Effectiveness of stimulation when oscillations and burst activity were abolished. A, B: Raster plot of a stimulation with a single -/+ 80 μA 1000 μs pulse at time point 0 applied to an MNU-treated retina showing oscillations and burst activity (A) and when oscillations and burst activity had been abolished by a prestimulus pulse sequence (10 pulses -/+ 80 μA 1000 μs, 1 Hz) (B). Every single vertical line marks a spike. In this cell, there were more stimulation-induced spikes after oscillations and burst activity had disappeared. C: The spike rate ratio was analyzed for a single pulse stimulation (-/+ 80 μA 1000 μs) prior to and after a prestimulus pulse sequence (10 pulses -/+ 80 μA 1000 μs, 1 Hz) that influenced the oscillations and burst activity. Each column represents the median, maximum, minimum, and the 50th percentile of the data for 6 MNU-treated retinas. Each mean is marked by a cross. On average, the stimulation was not more effective when oscillations and burst activity were abolished.

In Fig 5 we showed that ganglion cells that did not fire in bursts could be stimulated more efficiently than cells firing in bursts. We next tested, whether this was also true for ganglion cells that had been switched from the rhythmic bursting mode to the non-bursting mode by a prestimulus pulse sequence. Fig 7 shows the raster plots of a single pulse stimulation applied to an MNU-treated retina under control conditions (Fig 7A) and after oscillations and bursts had been abolished by a prestimulus pulse sequence (10 pulses -/+ 80 μA 1000 μs, 1 Hz) (Fig 7B). The increase of the spike rate induced by a single pulse stimulation is much more prominent after the prestimulus pulse sequence than before. However, we should point out that this behavior was only observed in a few ganglion cells. In the majority of ganglion cells, stimulation efficiency did not increase after oscillations and rhythmic burst activity had been abolished by the prestimulus pulse sequence (Fig 7C). The results showed in Figs 6, 7A and 7B were all obtained from the same piece of retina and the same recording electrode.

## Discussion

In the genetic mouse models for retinitis pigmentosa, *rd1* and *rd10*, the retina displays a spontaneous rhythmic electrical activity [12–15]. Here, we show for the first time a similar spontaneous activity in another model for retinal degeneration in which photoreceptor death has been induced pharmacologically by treatment with MNU. Although both cause and time course of degeneration are different from that of genetic models, similar rhythmic activity was recorded 9–11 days after injection of MNU when the ONL was strongly reduced in thickness as confirmed by OCT scans. It has been shown in other studies that at this time point most dendrites of horizontal cells and rod bipolar cells have been retracted, the axonal network of horizontal cells was thinned out, cell processes of hypertrophic Müller cells were extended, whereas other cells of the inner retina remained mostly unaffected [17, 18, 20, 22]. This status is similar to a retina of a 4 week old *rd10* mouse. Therefore, our results suggest that the oscillations are induced by the absence of photoreceptor input and corresponding functional changes within the inner retina rather than by massive anatomical remodeling of the inner retina. This is consistent with the findings of other studies showing that bipolar and amacrine cells are autonomously active and trigger RGC activity rhythmically when photoreceptors are degenerated in *rd1* retina [5, 21] or when photoreceptor input to the retinal network is pharmacologically blocked in wild type retina [23]. Also, the absence of oscillations in PNW2 *rd10* retinas and the occurrence of oscillations at PNW4 when loss of photoreceptors peaks, confirms the correlation between photoreceptor loss and oscillation development.

We observed in both genetically and pharmacologically induced degeneration models a strong reduction in the efficiency of electrical stimulation to trigger RGC activity compared to wild type retina. There seem to be two causes for this reduced stimulation efficiency. The first cause cannot be attributed to photoreceptor loss or the presence of rhythmic activity. Already at PNW2, when photoreceptors are still present and before oscillations and bursts appear, the stimulation efficiency in *rd10* is lower than in wild type. Thus, besides molecular changes [24] there are as well functional changes before photoreceptor degeneration begins. This may be correlated with reduced photoreceptor activity in *rd10* mice [7, 8]. However, there is a further decrease in stimulation efficiency at PNW4. Three observations suggest that this low stimulation efficiency rests on the presence of the abnormal rhythmic activity: 1. It occurs at the time when photoreceptors degenerate and oscillations and bursting activity develop. Once oscillations have been established at PNW4, stimulation efficiency does not decrease further despite the fact that degeneration proceeds. 2. The rhythmic activity was more stable and persistent in MNU-treated retinas than in *rd10* retinas. At the same time, the stimulation efficiency was even slightly lower in MNU-treated retinas than in *rd10* retinas. 3. We found a clear correlation between oscillations or burst activity and low stimulation efficiency. Stimulation of *rd10* neurons on electrodes that showed no oscillations was significantly more efficient than stimulation in presence of oscillations. Neurons firing in rhythmically occurring bursts were less excitable than neurons firing continuously. This was clearly observed in both *rd10* and MNU-treated retinas.

At present, we can only speculate about the reasons for this reduced stimulation efficiency. Ganglion cells remain unaffected even in the late phases of degeneration [2, 9]. Modulation of their synaptic input could cause changes leading to a lower excitability. Horizontal cells start to remodel at PNW4 [9–11] and thus their effect on bipolar cells could be altered [25]. Changes in the network of bipolar and amacrine cells could also influence the excitability of ganglion cells through increased inhibition [23, 26]. Furthermore, it is assumed that bursts originate by the interplay between bipolar and amacrine cells [14, 16, 21, 23, 27]. Their changed activity might play a crucial role in the excitability properties of ganglion cells.

In other retinal degeneration models like *rd1* mouse [12, 14, 21] and RCS rat [unpublished data] where oscillations and bursts were observed, thresholds for RGC stimulation are reported to be increased compared to the corresponding wild type [25, 28–35]. The stimulation thresholds of human RP retinas are elevated as well [36–40]. However nothing is known about the spontaneous activity in RP. Patients report the perception of light flashes which could reflect autonomous firing of bursts by retinal ganglion cells [5, 21, 27, 41–43]. In contrast, the P23H rat retina shows no elevated stimulation threshold, no oscillations and only a few cells show burst firing [44]. Taken together, all these reports confirm the assumption that the low stimulation efficiency correlates with the presence of rhythmic activity in form of oscillations and bursts.

An important result of our study is the possibility to abolish oscillations and burst activity by electrical stimulation. In some cases, the switch from oscillatory to normal activity was concomitant with an increase of stimulation efficiency. However, in the majority of cells we could not achieve a clear cut enhancement of the stimulation efficiency with this prestimulus pulse sequence approach, probably because the parameters which were chosen for the prestimulus pulse sequence treatment were not ideal. Optimal parameters will have to be determined in future studies. Nevertheless, these findings support the idea that it is possible to modulate spontaneous activity in the degenerated retina and that this modulation may help to increase the efficacy of electrical stimulation with implantable devices.

In summary, our results indicate that photoreceptor loss causes not only a change in spontaneous activity but also a change in excitability. The reduced excitability depends on the presence of abnormal rhythmic activity. However, it may be possible to modify this pathological activity until it resembles wild type activity. Modifications in currently applied stimulation protocols and paradigms used in today available retina implant technology may improve the stimulation efficiency. Further investigations are necessary and worthwhile to define optimal strategies for electrical stimulation in retinas with photoreceptor degenerations.
